# Antepartum Exposure to Greenness, Air Pollution, and Temperature and Outcomes of Preterm Infants

**DOI:** 10.1001/jamanetworkopen.2026.0102

**Published:** 2026-02-26

**Authors:** Alice Aveline, Nicole Bando, Mohammad Noaeen, Jie Yang, Thuy Mai Luu, Marc Beltempo, Abhay Lodha, Stephen J. Lye, Sarah D. McDonald, Charlene C. Nielsen, Alvaro R. Osornio-Vargas, David M. Stieb, Anne R. Synnes, Paul J. Villeneuve, Cheryl Battersby, Jeffrey R. Brook, Prakesh S. Shah

**Affiliations:** 1 Neonatal Medicine Research Group, Imperial College London, London, United Kingdom; 2 Department of Pediatrics, Mount Sinai Hospital, Toronto, Ontario, Canada; 3 Dalla Lana School of Public Health and the Department of Chemical Engineering and Applied Chemistry, University of Toronto, Toronto, Ontario, Canada; 4 Institute of Health Policy, Management and Evaluation, University of Toronto, Toronto, Ontario, Canada; 5 Department of Pediatrics, Centre Hospitalier Universitaire Sainte-Justine, Montreal, Quebec, Canada; 6 Division of Neonatology, Montreal Children’s Hospital, McGill University Health Centre, Montreal, Quebec, Canada; 7 Department of Pediatrics, McGill University, Montreal, Quebec, Canada; 8 Department of Epidemiology, Biostatistics, and Occupational Health, McGill University, Montreal, Quebec, Canada; 9 Department of Pediatrics, University of Calgary, Calgary, Alberta, Canada; 10 Lunenfeld-Tanenbaum Research Institute, Mount Sinai Hospital, Toronto, Ontario, Canada; 11 Department of Physiology, University of Toronto, Toronto, Ontario, Canada; 12 Department of Obstetrics and Gynecology, University of Toronto, Toronto, Ontario, Canada; 13 Department of Obstetrics and Gynecology, McMaster University, Hamilton, Ontario, Canada; 14 School of Public Health, University of Alberta, Edmonton, Alberta, Canada; 15 Department of Pediatrics, University of Alberta, Edmonton, Alberta, Canada; 16 Air Quality Health Effects Research Section, Health Canada, Ottawa, Ontario, Canada (Retired); 17 Department of Pediatrics, BC Women’s Hospital, University of British Columbia, Vancouver, British Columbia, Canada; 18 BC Children’s Hospital Research Institute, University of British Columbia, Vancouver, British Columbia, Canada; 19 Department of Neuroscience, Carleton University, Ottawa, Ontario, Canada; 20 Department of Pediatrics, University of Toronto, Toronto, Ontario, Canada

## Abstract

**Importance:**

Existing evidence shows that the environment is a risk factor in preterm birth. However, more evidence is needed about the association between environmental exposures and outcomes of preterm birth.

**Objective:**

To examine the association of antepartum exposure to individual and combined indices of greenness, air pollution, and extreme temperatures with outcomes of preterm infants.

**Design, Setting, and Participants:**

This cohort study used linked national databases of the Canadian Neonatal Network and Canadian Urban Environmental Health Research Consortium. The cohort consisted of infants born at 22 to 28 weeks plus 6 days’ gestation between January 1, 2010, and December 31, 2020, and treated in tertiary neonatal intensive care units (NICUs) across Canada. Data were analyzed between May 1 and September 15, 2024.

**Exposure:**

Indices summarizing levels of greenness, air pollutants, and ambient temperature at mothers’ residential postal code at birth.

**Main Outcome and Measure:**

Infant survival without major morbidity (SWMM) assessed at death or discharge from the NICU. Logistic regression models estimated associations between individual and combination of indices with SWMM.

**Results:**

A total of 14 748 infants (7965 males [54.0%]; mean [SD] gestational age, 26.1 [1.6] weeks; median [IQR] birth weight, 890 [720-1090] g) were included. The rate of SWMM was 32.1% (4737 of 14 748). Infants born to mothers who were exposed to high ozone levels had lower odds of SWMM vs infants with mothers exposed to low ozone levels (adjusted odds ratio [AOR], 0.83; 95% CI, 0.74-0.95). Infants with antepartum exposure to low temperature combined with either high levels of ozone (AOR, 0.76; 95% CI, 0.60-0.95) or low levels of greenness (AOR, 0.77; 95% CI, 0.60-0.99) or both low levels of greenness and high levels of ozone (AOR, 0.58; 95% CI, 0.43-0.77) had lower odds of SWMM than infants with mothers who were not exposed to these risk factors.

**Conclusions and Relevance:**

In this cohort study, antepartum exposure to high ozone and combined low temperatures and low levels of greenness or high ozone were associated with lower odds of SWMM in preterm infants. These findings suggest that the health outcomes of antepartum environmental exposure extend to neonatal outcomes of preterm infants.

## Key Points

**Question:**

Is antepartum exposure to green spaces, air pollutants, and extreme temperatures associated with the neonatal outcomes of preterm infants?

**Findings:**

In this cohort study of 14 748 extremely preterm infants that used data from linked national environmental and neonatal databases, maternal antepartum exposure to low levels of greenness, high levels of ozone, and low temperatures was associated with significantly lower odds of survival without major morbidity in infants compared with infants born to mothers who were unexposed.

**Meaning:**

The findings suggest that antepartum exposure to environmental stressors is associated with neonatal outcomes of infants born before 29 weeks’ gestation.

## Teaser

This cohort study investigates the association between prenatal exposure to environmental factors and outcomes among infants born between 22 and 28 weeks’ gestation in Canada.

## Introduction

The World Health Organization estimates that 23% of global deaths are caused by modifiable environmental factors.^1^ Increased risk of stillbirth,^2,3,4^ low birth weight,^3,4,5,6,7,8,9^ and preterm birth^3,4,6,7,8,9,10^ have been observed in women exposed to air pollution, lack of vegetation (greenness), and extreme temperatures. Data from full-term infants suggest that antepartum exposure to fine particulate matter with a diameter of 2.5 μm and 10 μm or less (PM_2.5_ and PM_10_) is associated with higher rates of assisted ventilation and antibiotic use in neonates.^11,12^ Antepartum exposure to higher ozone levels and PM_10_, although not PM_2.5_, has been linked to lower motor function.^13^ Antepartum exposure to extreme temperatures (both hot and cold) has been associated with reduced lung volumes in infants.^14^ Higher levels of environmental greenness during pregnancy have been linked to lower blood pressure levels in the first 4 days^15^ and higher developmental scores at 6 months in infants.^16^

Extremely preterm infants are at greater risk of death and major neonatal morbidities, including bronchopulmonary dysplasia, necrotizing enterocolitis, sepsis, severe brain injury (intraventricular hemorrhage and periventricular leukomalacia), and severe retinopathy of prematurity (ROP).^17,18^ However, data are lacking on the association between antepartum environmental exposures and neonatal outcomes of preterm infants. Understanding the individual and combined implications of these exposures for outcomes in infants born before 29 weeks’ gestation could help direct resources and support preventive interventions. Thus, our objective was to examine the association of antepartum exposure to individual and combined indices of greenness, air pollution, and extreme temperatures with outcomes of preterm infants. We sought to determine whether preterm infants with similar gestational age and birth weight characteristics had neonatal outcomes that differed as a function of antepartum environmental exposures.

## Methods

### Study Design and Participants

We conducted a retrospective cohort study using data from the Canadian Neonatal Network (CNN)^19^ linked to social and environmental exposure data from the Canadian Urban Environmental Health Research Consortium (CANUE), Canada’s environmental exposure data hub.^20,21^ The Mount Sinai Hospital Research Ethics Board and CNN Executive Committee approved this study and waived the informed consent requirement because of the retrospective nature of the study. We followed the Strengthening the Reporting of Observational Studies in Epidemiology (STROBE) reporting guideline.

Neonatal data on eligible admissions to neonatal intensive care units (NICUs) were collected from all 32 tertiary NICUs using a standardized manual.^22^ The database has been shown to be internally valid and consistent.^22,23^ The CANUE platform contains data on environmental features, such as greenness, air quality, weather, and neighborhood deprivation at a postal code level across Canada.^20^ Data from CNN and CANUE were linked deterministically using maternal residential postal code at infant’s birth.

Infants born between 22 and 28 weeks plus 6 days’ gestation between January 1, 2010, and December 31, 2020 (11 years), were included. Data were analyzed between May 1 and September 15, 2024. Infants were excluded if they were not provided life support at NICU admission; had major congenital anomalies^24^; had missing postal code data (and therefore environmental exposures could not be reliably inferred); or had an implausible birth weight.

### Exposure

We studied 3 categories of environmental exposures. Data on the level of greenness (averaged over the growing season and annually), air pollutants (monthly mean of ozone, nitrogen dioxide [NO_2_], PM_2.5_, and sulfur dioxide), and ambient temperature estimated at the mother’s residential postal code (on a monthly time scale, derived from hourly temperature data) were extracted from the CANUE database. Air pollution and temperature exposures were computed as monthly measures and averaged over the 9 months prior to birth; greenness was averaged over 12 months to smooth seasonal fluctuation. The air-quality domain included a monthly smoke variable (from the Canadian Optimized Statistical Smoke Exposure Model) to capture wildfire-related PM_2.5_ contribution. Details of the variables extracted are shown in eMethods 1 in Supplement 1. The data collection period covered the 9 months prior to birth, including the 8 to 14 weeks before conception. These data were converted into 4 indices: 1 greenness index, 2 air pollution indices, and 1 temperature index.

### Outcomes

The primary outcome was neonatal survival without major morbidity (SWMM) at death or discharge from the NICU. Infants who survived without major morbidity were compared with those who died or experienced major morbidity. Major neonatal morbidity was defined as any of the following: grade 3 or higher intraventricular hemorrhage^25^ or periventricular leukomalacia; bronchopulmonary dysplasia, defined as receipt of any respiratory support at 36 weeks of postmenstrual age or at the time of discharge to level 2 NICU, whichever occurred first; stage 3 or higher ROP in either eye^26^ or treated ROP; stage 2 or higher necrotizing enterocolitis^27^; or late-onset sepsis, defined as any blood and/or cerebrospinal fluid culture positive for bacteria, viruses, or fungi after 2 days of age. The secondary outcome was mortality prior to discharge.

### Statistical Analysis

We used principal component analysis (PCA) given the limitations of traditional regression models in assessing multiple exposures simultaneously.^28^ The environmental variables were prepared for PCA (eMethods 2 in Supplement 1). Patterns of missingness and levels of correlation were examined. The PCA analysis was performed using the FactoMineR package in R (R Project for Statistical Computing). We selected as many components as were required to account for at least 65% of the variance in the data. Environmental indices were categorized into tertiles to facilitate clinical interpretation.

Logistic regression was used to calculate the association between antepartum exposure and outcome. Following the directed acyclic graph (eFigure 1 in Supplement 1), the regression models included adjustment for 2 indices of neighborhood deprivation (eMethods 3 in Supplement 1), maternal diabetes, maternal substance use, maternal hypertension, gestational age, outborn status (birth outside a tertiary center), small for gestational age (below the 10th percentile), sex, severity of illness assessed by Score for Neonatal Acute Physiology II (SNAP-II; score range: 0-115, with higher scores indicating a more severely ill and physiologically unstable infant),^29^ birth weight, and birth epoch (coded as 2010-2013, 2014-2017, and 2018-2020) (eTable 1 in Supplement 1). Race and ethnicity data were not systematically collected and had considerable missing values; thus, they were not analyzed.

We assumed linear effects for each covariate. Robust SEs were used to correct for correlation within a hospital. Pairwise comparisons were conducted between each tertile of the environmental variables. Associations were considered statistically significant at 2-sided *P* < .05. Models were specified to estimate associations conditional on infant maturity and size at birth to control for baseline neonatal risk and were not interpreted as total causation.

To examine the impact of missingness, we conducted a sensitivity analysis using 25 multiply imputed datasets. We used the R package mice and pooled effect estimates using Rubin rules.^30^

## Results

**Figure 1.**
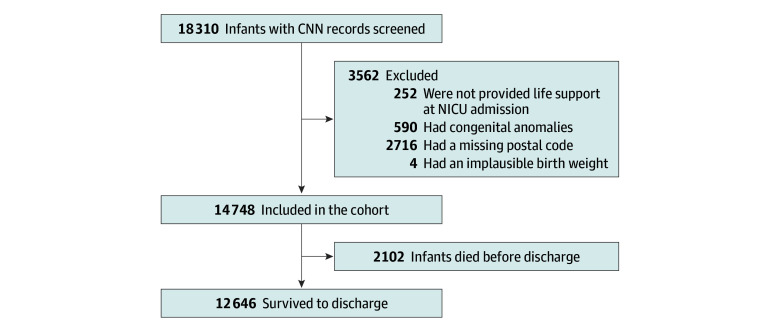
Flowchart of Study Cohort CNN indicates Canadian Neonatal Network; and NICU, neonatal intensive care unit.

Of the 18 310 infants with 22 to 28 weeks plus 6 days’ gestation admitted during the study period, 14 748 (80.6%) were included in the analysis (Figure 1). Among these infants, 6845 (46.4%) were born at 27 to 28 weeks’ gestation and 7903 (53.6%) were born before 27 weeks’ gestation (Table 1). This cohort had a mean (SD) gestational age of 26.1 (1.6) weeks and included 6764 females (45.9%) and 7965 males (54.0%). The median (IQR) birth weight was 890 (720-1090) g, and 1318 of 14 729 infants (8.9%) were small for gestational age. A total of 4737 infants (32.1%) survived without major morbidity and 2102 (14.3%) died before discharge.

**Table 1.** Key Characteristics of the Cohort (N = 14 748)

Characteristic	Cohort, No./total No. (%)
Maternal	
Diabetes	1533/13 966 (11.0)
Drug use	488/14 748 (3.3)
Hypertension	2277/14 333 (15.9)
Infant	
Gestational age, wk	
22-23	1010/14 748 (6.8)
24-26	6893/14 748 (46.7)
27-28	6845/14 748 (46.4)
Sex	
Female	6764/14 748 (45.9)
Male	7965/14 748 (54.0)
Missing data	19
Birth weight, median (IQR), g	890 (720-1090)
Missing data	5
SGA	1318/14 729 (8.9)
SNAP-II, median (IQR)^a^	14.0 (7.0-21.0)
Missing data	252
Birth epoch	
2010-2013	5570/14 748 (37.8)
2014-2017	5179/14 748 (35.1)
2018-2020	3999/14 748 (27.1)
Outcomes	
SWMM	4737/14 748 (32.1)
Any major morbidity	7909/14 748 (53.6)
Mortality	2102/14 748 (14.3)
BPD	6463/12 779 (50.6)
NEC	1155/14 707 (7.9)
Late-onset sepsis	3306/14 748 (22.4)
Grade 3/4 IVH	1813/14 041 (12.9)
≥Stage 3 ROP	1471/10 193 (14.4)

Abbreviations: BPD, bronchopulmonary dysplasia; IVH, intraventricular hemorrhage; NEC, necrotizing enterocolitis; ROP, retinopathy of prematurity; SGA, small for gestational age; SNAP-II, Score for Neonatal Acute Physiology II; SWMM, survival without any major morbidity.

^a^ SNAP-II score range: 0 to 115, with higher scores indicating a more severely ill and physiologically unstable infant.

### Principal Component Analysis

We conducted 1 PCA for greenness, air quality, and temperature. Each principal component formed an index that was included as an independent variable in later regression analyses. The first component of the greenness PCA accounted for 72.6% of the total variance, and therefore a single index to represent greenness was created. A higher greenness index score indicated greener neighborhoods.

Two indices were created to represent air quality. These components accounted for 49.7% (first index) and 27.7% (second index) of the total variance. High scores on the first index were associated with high levels of PM_2.5_, high levels of NO_2_, and low levels of smoke. High scores on the second index were predominantly associated with high levels of ozone. Hereafter, we use the terms *primary pollutants* and *ozone dominant* for these 2 indices; however, we recognize that the ozone dominant index also includes smaller components of other air pollutants.

The first component of the temperature PCA accounted for 65.1% of the total variance, and thus a single index was created. The temperature index summarized mean and extreme temperatures throughout the pregnancy; a higher score indicated warmer overall conditions. Further details on the PCA are provided in eMethods 2 in Supplement 1.

### Missingness Analysis

Greenness index values were available for 88.1%, air quality index values for 71.8%, and temperature index values for 90.8% of the cohort. In all cases, missingness of index values was not associated with SWMM (all *P* > .78). Of the covariates, none had more than 5% missing values; therefore, a complete case analysis was considered to be appropriate. A total of 5836 infants (39.6%) had missing data and were excluded from the regression analyses, whereas 8912 (60.4%) of the cohort had complete data. eTable 2 in Supplement 1 summarizes the background characteristics of infants with complete vs incomplete data.

### Association Between Individual Indices and Outcomes

Rates of SWMM stratified by tertiles of the exposure indices are shown in eFigure 2 in Supplement 1. Background characteristics of infants by levels of each exposure index are shown in eTable 3 in Supplement 1. Adjusted analyses are shown in Table 2 for the SWMM and mortality outcomes. Greenness and the primary pollutants (PM_2.5_, NO_2_, and smoke) air quality index were not associated with SWMM. High levels of the ozone dominant index were associated with lower odds of SWMM (highest to lowest tertiles: AOR, 0.83 [95% CI, 0.74-0.95]; *P* = .007; moderate to lowest tertiles: AOR, 0.89 [95% CI, 0.82-0.97]; *P* = .01). The association between the temperature index and SWMM was tenuous; only 1 contrast was statistically significant (moderate to lowest tertiles: AOR, 1.18 [95% CI, 1.07-1.31]; *P* = .001), indicating that moderate temperature was associated with higher odds of SWMM compared with lowest temperature. Analysis of the mortality outcome did not reveal any consistent pattern of associations (Table 2). The significant associations observed in the analysis were also significant in the sensitivity analysis of the imputed datasets (eTable 4 in Supplement 1).

**Table 2.** Association Between Environmental Exposures and Outcomes

Index and tertiles contrasted	RD, % (95% CI)	OR (95% CI)		*P* value for adjusted analysis
		Unadjusted	Adjusted^a^	
**SWMM**				
Greenness				
Highest to lowest	0.6 (−1.4 to 2.6)	1.10 (0.86 to 1.41)	1.05 (0.88 to 1.26)	.60
Moderate to lowest	2.5 (0.5 to 4.5)	1.14 (0.95 to 1.37)	1.12 (0.99 to 1.27)	.07
Highest to moderate	−1.9 (−3.9 to 0.1)	0.96 (0.83 to 1.11)	0.93 (0.83 to 1.05)	.27
Primary pollutants (PM_2.5_, NO_2_, and smoke)				
Highest to lowest	−0.3 (−2.6 to 1.8)	0.97 (0.71 to 1.32)	0.96 (0.77 to 1.20)	.74
Moderate to lowest	−0.2 (−2.5 to 1.9)	1.00 (0.86 to 1.15)	0.99 (0.90 to 1.08)	.74
Highest to moderate	−0.1 (−2.3 to 2.1)	0.97 (0.73 to 1.28)	0.95 (0.78 to 1.15)	.60
Ozone dominant				
Highest to lowest	−3.4 (−5.6 to 1.2)	0.87 (0.77 to 0.99)	0.83 (0.74 to 0.95)	.007
Moderate to lowest	−2.9 (−5.1 to −0.7)	0.92 (0.82 to 1.02)	0.89 (0.82 to 0.97)	.01
Highest to moderate	−0.5 (−2.7 to 1.7)	0.95 (0.87 to 1.04)	0.94 (0.84 to 1.04)	.20
Temperature				
Highest to lowest	2.2 (0.3 to 4.1)	1.18 (0.91 to 1.52)	1.14 (0.96 to 1.36)	.12
Moderate to lowest	2.6 (0.8 to 4.6)	1.14 (0.98 to 1.32)	1.18 (1.07 to 1.31)	.001
Highest to moderate	−0.4 (−2.5 to 1.5)	1.03 (0.90 to 1.19)	0.96 (0.85 to 1.10)	.60
**Mortality**				
Greenness				
Highest to lowest	0.6 (−0.9 to 2.1)	1.08 (0.89 to 1.31)	1.01 (0.84 to 1.22)	.91
Moderate to lowest	−0.4 (−1.8 to 1.0)	0.96 (0.85 to 1.09)	0.93 (0.83 to 1.05)	.23
Highest to moderate	1.0 (−0.4 to 2.4)	1.12 (0.99 to 1.27)	1.06 (0.91 to 1.24)	.44
Primary pollutants (PM_2.5_, NO_2_, and smoke)				
Highest to lowest	−1.7 (−3.3 to −0.1)	0.89 (0.74 to 1.08)	0.87 (0.73 to 1.02)	.09
Moderate to lowest	0.4 (−1.2 to 2.0)	1.03 (0.91 to 1.17)	1.00 (0.92 to 1.08)	.97
Highest to moderate	−2.1 (−3.7 to −0.5)	0.87 (0.77 to 0.97)	0.87 (0.76 to 0.99)	.04
Ozone dominant				
Highest to lowest	1.6 (0.0 to 3.2)	1.10 (0.91 to 1.33)	1.19 (0.99 to 1.44)	.07
Moderate to lowest	2.1 (0.5 to 3.7)	1.11 (0.92 to 1.34)	1.13 (0.96 to 1.34)	.14
Highest to moderate	−0.5 (−2.1 to 1.1)	0.99 (0.83 to 1.17)	0.95 (0.80 to 1.13)	.55
Temperature				
Highest to lowest	−0.3 (−1.7 to 1.1)	0.93 (0.76 to 1.13)	0.87 (0.71 to 1.08)	.21
Moderate to lowest	0.8 (−0.6 to 2.2)	0.98 (0.85 to 1.14)	1.04 (0.89 to 1.21)	.61
Highest to moderate	−1.1 (−2.5 to 0.3)	0.94 (0.82 to 1.09)	0.91 (0.76 to 1.08)	.28

Abbreviations: NO_2_, nitrogen dioxide; OR, odds ratio; PM_2.5_, fine particulate matter with a diameter of 2.5 μm or less; RD, risk difference; SWMM, survival without major morbidity.

^a^ ORs were calculated using logistic regression and adjusted for gestational age at birth, inborn or outborn status, birth weight, birth epoch, maternal diabetes, maternal antepartum drug use, maternal antepartum hypertension, small for gestational age, sex, Score for Neonatal Acute Physiology II, and neighborhood risk indices in tertiles.

### Association Between Combined Indices and Outcomes

**Figure 2.**
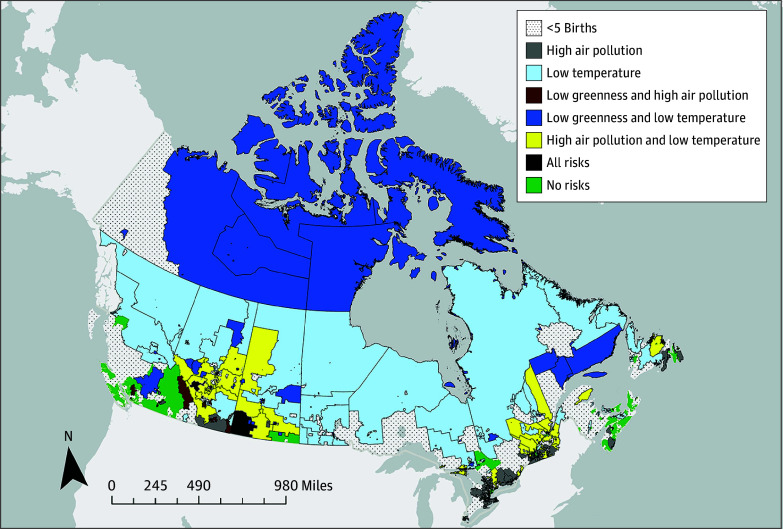
Map of Geographic Distribution of Environmental Risk Factors in Canada Environmental data for all analyses were based on the mother’s 6-digit postal code. The figure shows the levels of environmental variables averaged across all of the cohort births within a forward sortation area (3-digit postal code) and classified into tertiles. Forward sortation areas with fewer than 5 births were excluded to avoid identification of individuals.

Our analysis of individual indices identified 3 risk factors associated with lower odds of SWMM (low temperature, low greenness, and high ozone). We examined the impact of these combined risk factors using a logistic regression model that included an 8-level factor variable that coded for the combined presence or absence of each of these 3 risk factors. Table 3 reports the results of combinations of the environmental variables. Low temperature and low level of greenness (SWMM, 29.1%; AOR, 0.77 [95% CI, 0.60-0.99]; *P* = .04); low temperature and high level of ozone (SWMM, 31.5%; AOR, 0.76 [95% CI, 0.60-0.95]; *P* = .02); and low temperature, high level of ozone, and low level of greenness (SWMM, 24.6%; AOR, 0.58 [95% CI, 0.43-0.77]; *P* < .001) were the combinations associated with SWMM. The geographic distribution of these risk factors in the Canadian landscape is shown in Figure 2.

**Table 3.** Association Between Exposure to Combinations of Environmental Stressors and Odds of Survival Without Major Morbidity

Exposure	No. of infants^a^	SWMM, No. (%)	OR (95% CI)		*P* value for adjusted analysis
			Unadjusted	Adjusted^b^	
No risk	2648	927 (35.0)	1.00 [Reference]	1.00 [Reference]	NA
Low level of greenness only	1372	454 (33.1)	0.92 (0.75-1.12)	0.95 (0.81-1.12)	.55
High level of ozone only	1771	561 (31.7)	0.86 (0.75-1.00)	0.84 (0.73-0.96)	.01
Low temperature only	1388	459 (33.1)	0.92 (0.75-1.14)	0.90 (0.76-1.08)	.24
Low level of greenness and high levels of ozone	656	217 (33.1)	0.92 (0.65-1.30)	0.95 (0.71-1.27)	.73
Low temperature and low level of greenness	876	255 (29.1)	0.76 (0.56-1.04)	0.77 (0.60-0.99)	.04
Low temperature and high level of ozone	639	201 (31.5)	0.86 (0.67-1.09)	0.76 (0.60-0.95)	.02
Low temperature, high level of ozone, and low level of greenness	240	59 (24.6)	0.61 (0.41-0.90)	0.58 (0.43-0.77)	<.001

Abbreviations: NA, not applicable; OR, odds ratio; SWMM, survival without major morbidity.

^a^ Infants were classified into exposure groups as a function of whether they were in the lowest tertile of the greenness index, highest tertile for ozone, or lowest tertile for ambient temperature.

^b^ Adjusted for gestational age at birth, birth weight, birth epoch, maternal diabetes, maternal antepartum drug use, maternal hypertension, small for gestational age, sex, Score for Neonatal Acute Physiology II, and neighborhood risk indices in tertiles.

## Discussion

In this national cohort of infants with less than 29 weeks’ gestation, we identified that infants whose mothers were exposed to all 3 environmental risk factors (low levels of greenness, high levels of ozone, and low temperatures) had markedly lower rates of SWMM than infants whose mothers were not exposed to any of these risk factors. We also found that infants of mothers exposed to high levels of ozone had significantly lower rates of SWMM. Because gestational age and birth weight may partly mediate environmental associations, our estimates, adjusted for gestational age, represent associations with the environment that were not mediated by gestational age and likely underestimate any total effect of operating through preterm birth or lower birth weight.

We identified an association between the ozone dominant index and SWMM in extremely preterm infants. Exposure to ozone is associated with preterm birth^31^ and small for gestational age.^32,33^ Our report extends findings to neonatal outcomes of preterm infants. In the Canadian warm seasons, when ozone exposures are higher, concentrations also tend to be highest in the suburbs and downwind of cities and lower closer to city centers where NO_2_ is higher. It is possible that the associations observed here may be attributed to some other features of suburban and near urban living (eg, more sedentary lifestyles, longer commutes, and less time for healthy eating). The ozone dominant index also includes a small positive weighting for PM_2.5_ (ie, higher levels of PM_2.5_ are associated with higher index levels) and a small negative weighting for smoke and NO_2_ (ie, lower levels of smoke and NO_2_ are associated with higher index levels). It is possible that the association we observed between the ozone dominant air quality index and SWMM was due to the joint properties of ozone, other co-occurring oxidant gases, and similar oxidizing components of PM_2.5_ rather than ozone alone.

In our cohort, the primary pollutants index related to PM_2.5_ and NO_2_ was not associated with SWMM, despite known association between these pollutants and birth outcomes.^34^ However, we suggest caution in interpretation, as we are not suggesting that those pollutants are harmless, only that we did not detect an association in this study. Canada ranks in the top 15 countries with the lowest levels of PM_2.5_,^35^ and annualized levels of NO_2_ have met Canadian Ambient Air Quality Standards in recent years.^36^ The lack of association may reflect the relatively benign Canadian conditions for the pollutants that dominate this index. Conversely, the geospatial pattern of the primary pollutants air quality index may also reflect other unmeasured features of urban form, such as walkability or better access to health care.

Most studies of the association of environmental factors on birth outcomes have focused on a single environmental exposure; however, most exposures are co-occurring for an individual. For example, NO_2_ and PM_2.5_ tend to be higher in winter as thermal inversions trap cold air and pollution closer to the ground.^37^ Similarly, vegetation reduces temperature through shading and transpiration^38^ but also impedes air movement, may reduce the dispersion of air pollution, and prevents the introduction of air pollution.^39^ Thus, mechanistically, these variables may modify each other’s independent outcomes or affect their potency in combination. We identified an association between antepartum exposure to low temperatures and lower rates of SWMM when accompanied by other exposures. This finding is consistent with reports of an association between antepartum exposure to cold temperatures and preterm birth^34,40^ and lung function in term-born infants.^14^ Studies have also reported an association between high temperatures and preterm birth, low birth weight, and stillbirth.^40,41^ We found no evidence of an association between high temperatures and outcomes. It is possible that the impact of high temperature may be reduced in populations with widespread air conditioning use, such as in Canada where 61% of the population has household air conditioning^42,43^; however, we do not have individual data. Further studies are warranted.

The associations between air quality, cold temperatures, and SWMM that we observed may be attributed to a number of biological mechanisms, including systemic inflammatory response^44,45,46^ and oxidative stress.^47,48,49,50,51^ Each environmental domain may change neonatal outcomes via distinct mechanism. Air pollution affects the synthesis, activity, and function of proteins in the placenta, leading to alterations in fetal genetic expression and transcriptional regulation.^52,53,54,55^ Increased blood viscosity from cold temperatures may reduce uterine blood flow, adversely impacting fetal development.^56^ To elucidate this relationship, other methods—such as joint models with penalization, supervised single-index models, other unsupervised reductions, and clustering—can emphasize different aspects of correlated exposures; however, these methods are beyond the scope of this study. Because PCA is unsupervised, we present the indices as transparent summaries rather than outcome-optimized constructs. Future work may opt to apply complementary frameworks to triangulate these findings.

### Strengths and Limitations

A strength of this study was the use of a nationally representative cohort with data from 2 linked unique, comprehensive databases. The breadth of the CNN database allowed us to control for clinical variables, and the multipollutant data available within the CANUE platform allowed us to investigate the association of simultaneous exposures.

The study also had limitations. First, we used a retrospective cohort design, which is subject to potential bias from residual confounding. We do not interpret these results causally. For example, the significant association between air pollution and SWMM may correspond to urban centers with better proximity to advanced neonatal intensive care. Measurement bias and residual confounding may exist given that socioeconomic factors are difficult to measure accurately and completely, particularly when using area-level measures of deprivation.^57,58^ We did not adjust for the individual NICU because of concerns about data sparsity and overfitting. We recognize that there may be differences in rates of SWMM due to differences in clinical practices between NICUs.

Second, the measurement of exposure status for environmental variables is complex. Pollutants may be measured at different temporal frequencies, spatial resolutions and locations, creating the potential for estimation error when techniques are used to interpolate between data points. Our approach of averaging levels throughout the pregnancy targeted chronic exposure and thus attenuated short-lived episodes (eg, wildfire). Although a monthly smoke metric contributed to the primary pollutants index, acute peaks are likely underrepresented and any resulting misclassification would generally bias associations toward the null. In addition, we measured area-level exposures derived from the residential neighborhood and not personal exposures. We assumed that women spent the majority of time at home; however, it is possible that women were working and commuting for some part of their day and were exposed to different exposures. Personal exposure to air pollutants increases with ventilation and the amount of time spent outdoors,^59^ factors that are likely to vary even within a population. Hoek et al^60^ reported a high level of correlation between air pollution assessed at the residential address and exposure estimated, taking account of the time spent in different locations and activities. However, we may have minimal exposure misclassification.

Third, we assumed constant residential location during the 9 months of exposure. There is a possibility of measurement error; however, only a small number of mothers relocate substantial distances during pregnancy.^61^ Fourth, we did not use the fetus-at-risk approach as the data were not available and we aimed to evaluate neonatal outcomes, not pregnancy outcomes. Fifth, we did not have race and ethnicity data for our cohort. Sixth, regarding temperature exposure, we combined chronic (monthly means) and acute (extreme days) temperatures. This combination may obscure distinct physiological pathways, and future studies should consider modeling acute and chronic temperatures separately.

## Conclusions

In this cohort study of extremely preterm infants, low levels of greenness, high levels of ozone, and low temperatures were associated with lower odds of SWMM in infants with antepartum exposure compared with infants born to mothers without antepartum exposure. Exposure to low temperatures combined with low levels of greenness or high ozone was also associated with lower odds of SWMM. It is concerning that, in a high-income country such as Canada, associations between environmental stressors and neonatal outcomes are still detectable. Further studies in other populations are needed as well as studies evaluating long-term implications for childhood growth and respiratory and neurodevelopmental outcomes.

## Notes

Supplement 1. **eMethods 1.** Measures of Exposure to Environmental Stressors **eMethods 2.** Principal Component Analysis **eMethods 3.** Creation of Neighborhood Deprivation Indices **eTable 1.** Structure of Covariate Variables **eTable 2.** Background Characteristics of Infants With Complete vs Incomplete Data **eTable 3.** Background Characteristics of Infants by Level of Environmental Exposure Index **eTable 4.** Results From the Sensitivity Analysis Using Multiple Imputation **eFigure 1.** Directed Acyclic Graph Summarizing the Causal Relationships Associated With the Exposure and Outcome **eFigure 2.** Rates of Survival Without Major Morbidity Stratified by Tertiles of the Exposure Indices

Supplement 2.
Nonauthor Collaborators

Supplement 3.
Data Sharing Statement
