# Supplementary material for: Antepartum Exposure to Greenness, Air Pollution, and Temperature and Outcomes of Preterm Infants
Source: JAMA Netw Open. 2026 Feb 26;9(2):e260102. doi: 10.1001/jamanetworkopen.2026.0102 (PMC12947023; doi:10.1001/jamanetworkopen.2026.0102)
Supplement: Supplement 1. — eMethods 1. Measures of Exposure to Environmental Stressors eMethods 2. Principal Component Analysis eMethods 3. Creation of Neighborhood Deprivation Indices eTable 1. Structure of Covariate Variables eTable 2. Background Characteristics of Infants With Complete vs Incomplete Data eTable 3. Background Characteristics of Infants by Level of Environmental Exposure Index eTable 4. Results From the Sensitivity Analysis Using Multiple Imputation eFigure 1. Directed Acyclic Graph Summarizing the Causal Relationships Associated With the Exposure and Outcome eFigure 2. Rates of Survival Without Major Morbidity Stratified by Tertiles of the Exposure Indices [file jamanetwopen-e260102-s001.pdf]

Supplemental Online Content

Aveline A, Bando N, Noaeen M, et al; on behalf of Canadian Neonatal Network Investigators and Canadian Urban Environmental Health Research Consortium. Antepartum exposure to greenness, air pollution, and temperature and outcomes of preterm neonates. *JAMA Netw Open*. 2026;9(2):e260102. doi:10.1001/jamanetworkopen.2026.0102

eMethods 1. Measures of Exposure to Environmental Stressors

eMethods 2. Principal Component Analysis

eMethods 3. Creation of Neighborhood Deprivation Indices

eTable 1. Structure of Covariate Variables

eTable 2. Background Characteristics of Infants With Complete vs Incomplete Data

eTable 3. Background Characteristics of Infants by Level of Environmental Exposure Index

eTable 4. Results From the Sensitivity Analysis Using Multiple Imputation

eFigure 1. Directed Acyclic Graph Summarizing the Causal Relationships Associated With the Exposure and Outcome

eFigure 2. Rates of Survival Without Major Morbidity Stratified by Tertiles of the Exposure Indices

This supplemental material has been provided by the authors to give readers additional information about their work.

eMethods 1: Measures of exposure to environmental stressors

Air pollution and temperature data were averaged over the 9 months prior to birth. Greenness data were averaged over the 12 months prior to birth to remove seasonality effects, since we were primarily interested in the greenness of the neighborhood on average over the year, rather than the influence of seasonal factors.

**Exposure category**	**Exposure**	**Source/Description**	**Additional Description**
Air pollution	Nitrogen Dioxide	Land use regression model developed using 2006 national air pollution surveillance (NAPS) monitoring data. Model development described in Hystad P, Setton E, Cervantes A, Poplawski K, Deschenes S, Brauer M, et al. 2011. Creating National Air Pollution Models for Population Exposure Assessment in Canada. Environ. Health Perspect. 119:1123–1129; doi:10.1289/ehp.1002976.	Monthly average concentration (ppb)
		GEM-MACH NO2 developed by Environment and Climate Change Canada's (ECCC)	Monthly average concentration (ppb)
	Ozone	Ground-level ozone (O3) concentrations developed by Environment and Climate Change Canada (ECCC) estimated with CHRONOS model from 2002 to 2009, and with GEM-MACH model from 2010 to 2021	Monthly average concentration (ppb)
			Monthly average of the highest rolling 8-hour average per day (ppb)
		GEM-MACH ozone (O3) developed by Environment and Climate Change Canada's (ECCC)	Monthly average concentration (ppb)
	Fine particulate matter	Ground-level fine particulate matter (PM2.5) estimates over North America with ground-monitor based adjustment	Monthly average PM2.5 v2 concentration (ug/m3)
		Ground-level fine particulate matter (PM2.5) global estimates with ground-monitor based adjustment	Monthly average PM2.5 v5 concentration (ug/m3)
	Smoke	The Canadian Optimized Statistical Smoke Model (CanOSSEM) developed by the Environmental Health Services of the BC Centre for Disease Control (Hazard Mapping System- National Oceanic)	Monthly average PM2.5 concentration (ug/m3)
	Sulphur Dioxide	GEM-MACH ground-level sulfur dioxide (SO2) concentrations estimated from the Ozone Monitoring Instrument (OMI) satellite data using SO2 profiles from the GEM-MACH model over North America, provided by Environment and Climate Change Canada	Monthly average concentration (ppb)
Temperature	Maximum temperature in month	Hourly maximum temperature data was provided by Natural Resources Canada	Monthly maximum temperature
	Minimum temperature in month	Hourly minimum temperature data was provided by Natural Resources Canada	Monthly minimum temperature
	Number of days in month with maximum temperature at 30 degrees Celsius or above	Hourly maximum temperature data was provided by Natural Resources Canada	-
	Number of days in month with	Hourly maximum temperature data was provided by Natural Resources Canada	-

© 2026 Aveline A et al. *JAMA Network Open*

Exposure category	Exposure	Source/Description	Additional Description
	maximum temperature at 25 degrees Celsius or above		
	Number of days in month with minimum temperature at 0 degrees Celsius or below	Hourly minimum temperature data was provided by Natural Resources Canada	-
	Number of days in month with minimum temperature at minus 15 degrees Celsius or below	Hourly minimum temperature data was provided by Natural Resources Canada	-
	Number of days in month with minimum temperature at minus 30 degrees Celsius or below	Hourly minimum temperature data was provided by Natural Resources Canada	-
Greenness	Annual average NDVI	USGS LandSat 5 and 8 satellites data, accessed via Google Earth Engine	Annual average normalised difference vegetation index (NDVI) at postcode
			Average of annual average NDVI within 100 m
			Average of annual average NDVI within 250 m
			Average of annual average NDVI within 500 m
			Average of annual average NDVI within 1 km
	Growing season average NDVI		Growing season average NDVI at postcode
			Average of growing season average NDVI within 100 m
			Average of growing season average NDVI within 250 m
			Average of growing season average NDVI within 500 m
			Average of growing season average NDVI within 1 km

© 2026 Aveline A et al. *JAMA Network Open*

eMethods 2: Principal Component Analysis

Data preparation for PCA

Where an exposure was measured by indices from different sources, e.g., Land Use Regression and Global Environmental Multi-scale - Modelling Air Quality and Chemistry (GEM-MACH), and only one source had high levels of missingness, then the heavily missing variable was excluded from further analysis. Where exposure was measured by indices from different sources and both had significant levels of non-overlapping missingness, these measures were combined with preference given to values from the variable with the fewest missing values. Variables showing substantial skew were transformed using log, root or reciprocal transformations.

Greenness: Measures of greenness were strongly intercorrelated (minimum correlation = .47) and had completely overlapping patterns of missingness. Therefore, four measures of greenness were included as input into the PCA analysis (annual average greenness at the postcode and at 1km around the postcode, growing season greenness at the postcode and at 1km around the postcode).

Air quality: The two measures of NO_2 were highly correlated (r=0.81) and had high levels of missingness (34.9% and 54.5%), but with limited overlap in missingness, so they were combined into a single measure of NO_2 exposure. The Land Use Regression (LUR) measure was used where this was available, otherwise the GEM-MACH measure.
The three measures of ozone showed high levels of missingness (52.2%-68.5%). Missingness completely overlapped for the two ozone measures and they were highly correlated (r=0.89). The GEM-MACH measure was combined with the ground-level average of the highest rolling 8-hour measure. The ground-level measure was used where this was available, otherwise the GEM-MACH measure.
SO_2 measure was 54.5% missing and made little contribution to the first two principal components, therefore SO_2 was excluded from the final PCA analysis.

Temperature: All temperature variables were 9.2% missing with complete overlap.
The number of days above 30°C was highly correlated with the number of days above 25°C (r=0.83). Days above 25°C was removed from further analyses as it showed higher levels of correlation with other temperature variables than days above 30°C.
The number of days below -30°C was highly correlated with the number of days below -15°C (r=0.67). Days below -30°C was excluded from further analysis as it was more highly skewed than days below -15°C.
Maximum monthly temperature was highly correlated with the mode of daily maximum temperature (r=0.90) and the same was true for minimum temperatures (r=0.91). The mode measures were excluded from further analysis.

© 2026 Aveline A et al. *JAMA Network Open*

Missing data

60.4% of the cohort had complete data for all covariates and all environmental indices. As several environmental variables exhibited substantial levels of missingness, we visualised patterns of missingness to see in which variables missingness commonly co-occurred.

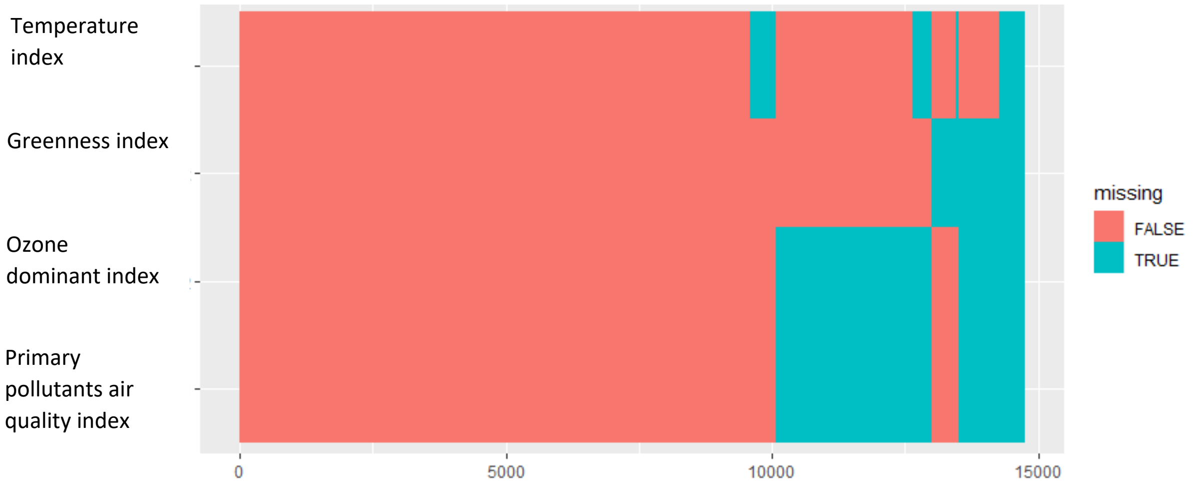

A complete case analysis will, in general, produce a biased estimate of the odds ratio and associated confidence interval for a binary exposure when missingness depends on both the outcome and exposure.[1] To determine whether multiple imputation for missing data was required, we examined whether the value of any covariate or its missingness (a binary variable) was associated with the outcome (survival without major morbidity) using regression models. Our results demonstrated that a complete case analysis was appropriate because no variables with substantial missingness had missingness that was associated with the outcome.

[1]Jonathan W. Bartlett, Ofer Harel, James R. Carpenter, Asymptotically Unbiased Estimation of Exposure Odds Ratios in Complete Records Logistic Regression, *American Journal of Epidemiology*, Volume 182, Issue 8, 15 October 2015, Pages 730–736, https://doi.org/10.1093/aje/kwv114

© 2026 Aveline A et al. *JAMA Network Open*

Results of the principal component analysis

Contribution graphs on the left-hand side of the page: Size and color of the circle represents the contribution of each of the original measures to the principal component(s) shown to the right.

PCA graphs on the right-hand side of the page*:* Positively correlated variables are grouped together. Negatively correlated variables are positioned on opposite sides of the plot origin (opposed quadrants). Variables that are close to the center of the plot are less important for the first components.

Greenness index

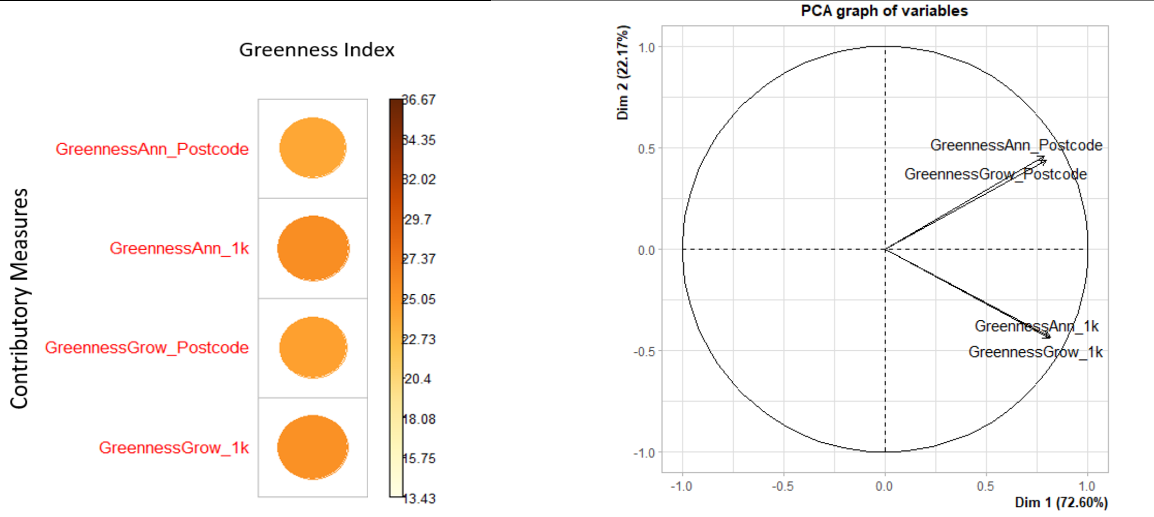

Air quality indices

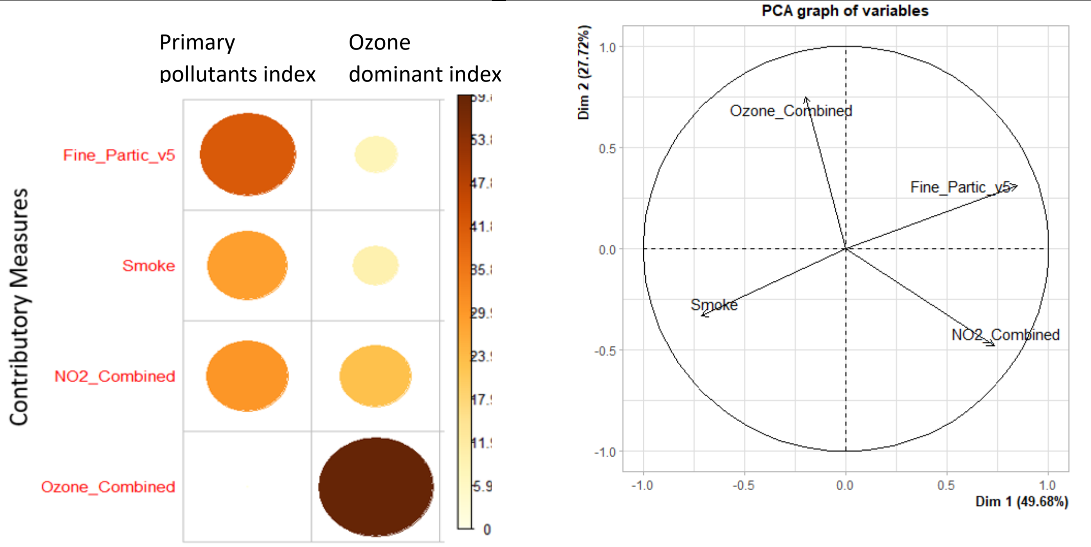

© 2026 Aveline A et al. *JAMA Network Open*

Temperature index

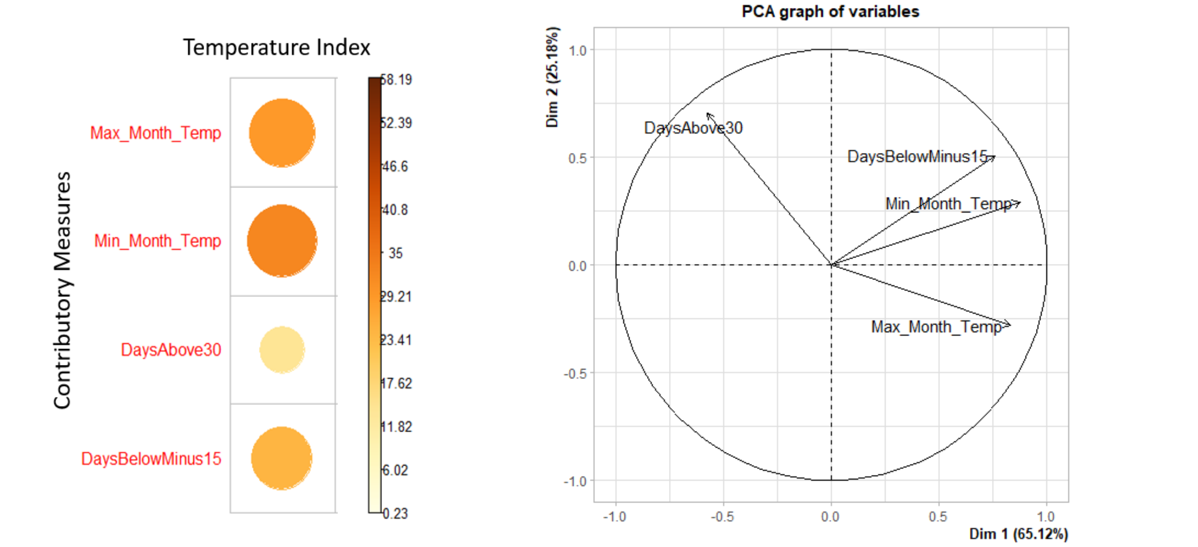

Key to variable names

Contributory measure	Description
GreennessAnn_Postcode	Annual average normalised difference vegetation index (NDVI) at postcode
GreennessAnn_1k	Average of annual average NDVI within 1 km
GreennessGrow_Postcode	Growing season average NDVI at postcode
GreennessGrow_1k	Average of growing season average NDVI within 1 km
Fine_Partic_v5	Ground-level fine particulate matter ($PM_{2.5}$) global estimates with ground-monitor based adjustment. Monthly average concentration (ug/m^3)
Smoke	The Canadian Optimized Statistical Smoke Model (CanOSSEM) developed by the Environmental Health Services of the BC Centre for Disease Control (Hazard Mapping System- National Oceanic). Monthly average $PM_{2.5}$ concentration (ug/m^3)
NO2_Combined	GEM-MACH NO_2 developed by Environment and Climate Change Canada's (ECCC). Monthly average concentration (ppb).<br>If unavailable, Land use regression model developed using 2006 national air pollution surveillance (NAPS) monitoring data. Model development described in Hystad P, Setton E, Cervantes A, Poplawski K, Deschenes S, Brauer M, et al. 2011. Creating National Air Pollution Models for Population Exposure Assessment in Canada. Environ. Health Perspect. 119:1123–1129; doi:10.1289/ehp.1002976. Monthly average concentration (ppb)
Ozone_Combined	Ground-level ozone (O_3) concentrations developed by Environment and Climate Change Canada (ECCC) estimated with GEM-MACH model. Monthly average of the highest rolling 8-hour average per day (ppb).<br>If unavailable, GEM-MACH ozone (O_3) developed by Environment and Climate Change Canada's (ECCC). Monthly average of the average per day (ppb).

© 2026 Aveline A et al. *JAMA Network Open*

Max_Month_Temp	Monthly average of hourly maximum temperature data provided by Natural Resources Canada
Min_Month_Temp	Monthly average of hourly minimum temperature data provided by Natural Resources Canada
DaysAbove30	Number of days in month with maximum temperature at 30 degrees Celsius or above
DaysBelowMinus15	Number of days in month with minimum temperature at minus 15 degrees Celsius or below

Summary statistics for the constituents of the indices

	**Median (IQR)**		
**Individual Measure**	Lowest tertile	Moderate tertile	Highest tertile
**Greenness index**			
GreennessAnn_Postcode	0.20 (0.14-0.26)	0.31 (0.26-0.37)	0.45 (0.38-0.53)
GreennessAnn_1k	0.31 (0.26-0.34)	0.39 (0.35-0.43)	0.49 (0.44-0.56)
GreennessGrow_Postcode	0.24 (0.18-0.30)	0.34 (0.29-0.39)	0.48 (0.41-0.55)
GreennessGrow_1k	0.34 (0.30-0.38)	0.42 (0.38-0.46)	0.54 (0.48-0.60)
**Primary pollutants air quality index**			
Fine_Partic_v5	5.87 (5.12-6.48)	7.30 (6.62-7.86)	8.64 (7.97-9.42)
Smoke	6.78 (6.49-7.08)	7.36 (7.03-7.76)	8.06 (7.60-8.90)
NO2_LUR	4.63 (2.69-6.67)	9.25 (6.45-12.72)	14.64 (11.46-17.64)
NO2_GEM	2.76 (1.23-4.77)	8.42 (6.10-10.92)	13.28 (9.50-15.83)
Ozone_High8	33.55 (30.91-36.59)	35.43 (31.12-39.18)	33.25 (30.52-36.20)
Ozone_GEM	29.10 (26.95-31.12)	25.79 (23.20-27.79)	23.19 (20.77-25.62)
**Ozone dominant index**			
Fine_Partic_v5	6.57 (5.59-7.74)	7.14 (6.10-8.28)	7.73 (6.82-8.66)
Smoke	7.10 (6.64-7.59)	7.29 (6.83-7.83)	7.67 (7.16-8.34)
NO2_LUR	13.47 (6.89-17.64)	10.04 (5.28-14.29)	7.70 (5.17-10.68)
NO2_GEM	12.10 (8.58-15.32)	6.37 (3.12-9.23)	3.83 (2.04-6.41)
Ozone_High8	29.72 (27.20-31.78)	33.75 (31.96-35.41)	38.20 (36.06-40.46)
Ozone_GEM	22.71 (20.20-24.39)	27.07 (25.82-28.46)	30.41 (28.87-29.45)
**Temperature index**			
Max_Month_Temp	17.23 (15.68, 18.52)	20.57 (18.94, 22.07)	24.19 (22.73, 25.53)
Min_Month_Temp	-12.33 (-15.29, -10.20)	-6.14 (-7.83, -4.30)	-0.92 (-2.99, 1.13)
DaysAbove30	0.22 (0.00-0.44)	0.44 (0.11-0.89)	1.33 (0.67-2.11)
DaysBelowMinus15	4.89 (3.33-6.78)	1.56 (0.55-2.67)	0.33 (0.00-0.89)

© 2026 Aveline A et al. *JAMA Network Open*

eMethods 3: Creation of neighborhood deprivation indices

We used the same PCA method that were used to create the environmental exposure indices to create two indices of neighborhood deprivation. The variables considered for inclusion in neighborhood deprivation indices are shown below. These variables were combined into two indices, accounting for 38% and 32% of the total variance respectively. For each of the two indices, the index values were categorized into three categories of deprivation: high, medium and low.

Variables considered for inclusion in neighborhood deprivation indices
The unit of spatial resolution for the indices is the dissemination area. There may be many postcodes within a single dissemination area – these will have the same index values. Canadian Marginalization Index (CAN-Marg), indexed to DMTI Spatial Inc. postcodes, were provided by CANUE.

Index name	Sub-index	Dimensions of deprivation captured by index	Timepoint of assessment
Canadian marginalization index	Residential instability	▪ Proportion of dwellings that are apartment buildings<br>▪ Proportion of persons living alone<br>▪ Proportion of dwellings that are not owned<br>▪ Proportion of movers within the last 5 years<br>▪ Proportion of the population that is not married/common-law<br>▪ Median household income	2011 and 2016
	Situational vulnerability	▪ Proportion of the population identified as indigenous<br>▪ Proportion of homes needing major repairs<br>▪ Proportion of the population aged 25-64 without a high school diploma<br>▪ Proportion of single parent families<br>▪ Median dollar value of dwelling	
	Economic dependency	▪ Proportion of population participating in the labour force (aged 15 and older)<br>▪ Proportion of the population aged 65 and older<br>▪ Ratio of employment to population<br>▪ Population aged under 15 years or over 64 years<br>▪ Proportion of population receiving government transfer payments	
	Ethno-cultural composition	▪ Proportion of the population that is foreign born<br>▪ Proportion of the population self-identified as a visible minority<br>▪ Proportion of the population with no knowledge of either official language<br>▪ Average number of persons per room<br>▪ Proportion of the population who are recent immigrants	

For further information about these indices, see the references below.

[1] Matheson et al. "Development of the Canadian Marginalization Index: a new tool for the study of inequality." Canadian Journal of Public Health, 2012;103(Suppl. 2):S12-S16.

© 2026 Aveline A et al. *JAMA Network Open*

[2] CanMap Postal Code Suite v2016.3. [Computer file] Markham: DMTI Spatial Inc., 2016.
[3] CanMap Postal Code Suite v2015.3. [computer file] Markham: DMTI Spatial Inc., 2015.

Weighting of the index components

Index 1 high values are associated with:

- High scores on CMI dependency
- Low scores on CMI ethnicity

Index 2 high values are associated with:

- High scores on CMI instability
- Low scores on CMI deprivation

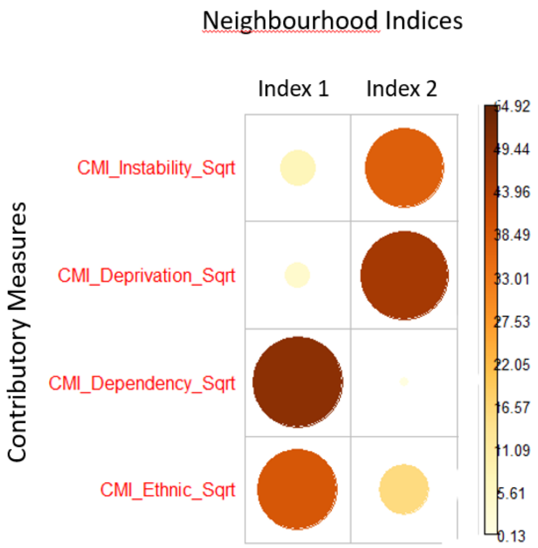

© 2026 Aveline A et al. *JAMA Network Open*

eTable 1: Structure of covariate variables

Variables adjusted for	Structure in regression model
Indices of neighborhood deprivation	Ordinal variable coded in three categories: high deprivation, moderate deprivation, low deprivation
Maternal diabetes	Binary (diabetic vs not diabetic)
Maternal substance use	Binary (substance user vs not substance user)
Maternal hypertension	Binary (hypertensive vs not hypertensive)
Gestational age	Ordinal variable coded in categories corresponding to completed full weeks of pregnancy i.e. 23, 24 etc
Outborn (birth outside a tertiary center),	Binary (birth outside a tertiary center vs birth in a tertiary center)
Small for gestational age (below the 10th centile),	Binary (small for gestational age vs not small for gestational age)
Infant sex	Binary (male vs female)
Severity of illness assessed by Score for Neonatal Acute Physiology-II (SNAP-II),	Binary (≤20 or >20)
Birthweight	Continuous variable (in grams)
Birth epoch	Ordinal variable coded in three categories: 2010-2013, 2014-2017 and 2018-2020

© 2026 Aveline A et al. *JAMA Network Open*

eTable 2: Background characteristics of infants with complete vs incomplete data

	Incomplete (N=5,836)	Complete (N=8,912)
**Gestational age at birth (weeks)**		
22-23	397 (6.8%)	613 (6.9%)
24-26	2,746 (47.1%)	4,147 (46.5%)
27-28	2,693 (46.1%)	4,152 (46.6%)
**Sex**		
Male	3,206 (54.9%)	4,759 (53.4%)
Female	2,611 (44.7%)	4,153 (46.6%)
Missing	19 (0.3%)	0 (0%)
**Birthweight (grams)**		
Median [Q1, Q3]	890 [720, 1,090]	890 [720, 1,090]
Missing	5 (0.1%)	0 (0%)
**Small for gestational age**		
Not SGA	5,316 (91.1%)	8,095 (90.8%)
SGA	501 (8.6%)	817 (9.2%)
Missing	19 (0.3%)	0 (0%)
**Snap II Score**		
Median [Q1, Q3]	14.0 [7.00, 22.0]	14.0 [7.00, 21.0]
Missing	106 (1.8%)	146 (1.6%)
**Birth year**		
2010-2013	2,090 (35.8%)	3,480 (39.0%)
2014-2017	1,879 (32.2%)	3,300 (37.0%)
2018-2020	1,867 (32.0%)	2,132 (23.9%)

© 2026 Aveline A et al. *JAMA Network Open*

eTable 3: Background characteristics of infants by level of environmental exposure index

3a. Greenness Index

	Low (N=2,929)	Medium (N=3,008)	High (N=2,975)
**Gestational age at birth (weeks)**			
22-23	191 (6.5%)	219 (7.3%)	203 (6.8%)
24-26	1,381 (47.1%)	1,415 (47.0%)	1,351 (45.4%)
27-28	1,357 (46.3%)	1,374 (45.7%)	1,421 (47.8%)
**Sex**			
Male	1,556 (53.1%)	1,607 (53.4%)	1,596 (53.6%)
Female	1,373 (46.9%)	1,401 (46.6%)	1,379 (46.4%)
**Birthweight**			
Median [Q1, Q3]	885 [720, 1,070]	880 [720, 1,080]	910 [730, 1,110]
**Small for gestational age**			
Not SGA	2,653 (90.6%)	2,721 (90.5%)	2,721 (91.5%)
SGA	276 (9.4%)	287 (9.5%)	254 (8.5%)
**Snap II Score**			
Median [Q1, Q3]	14.0 [7.00, 21.0]	14.0 [7.00, 21.0]	14.0 [7.00, 21.0]
Missing	52 (1.8%)	42 (1.4%)	52 (1.7%)
**Birth year**			
2010-2013	1,250 (42.7%)	1,201 (39.9%)	1,029 (34.6%)
2014-2017	1,000 (34.1%)	1,131 (37.6%)	1,169 (39.3%)
2018-2020	679 (23.2%)	676 (22.5%)	777 (26.1%)

© 2026 Aveline A et al. *JAMA Network Open*

3b. Primary Pollutants Air Quality Index

	Low (N=2,853)	Medium (N=2,988)	High (N=3,071)
**Gestational age at birth (weeks)**			
22-23	191 (6.7%)	223 (7.5%)	199 (6.5%)
24-26	1,274 (44.7%)	1,423 (47.6%)	1,450 (47.2%)
27-28	1,388 (48.7%)	1,342 (44.9%)	1,422 (46.3%)
**Sex**			
Male	1,486 (52.1%)	1,638 (54.8%)	1,635 (53.2%)
Female	1,367 (47.9%)	1,350 (45.2%)	1,436 (46.8%)
**Birthweight**			
Median [Q1, Q3]	910 [730, 1,110]	890 [710, 1,080]	880 [723, 1,070]
**Small for gestational age**			
Not SGA	2,590 (90.8%)	2,716 (90.9%)	2,789 (90.8%)
SGA	263 (9.2%)	272 (9.1%)	282 (9.2%)
**Snap II Score**			
Median [Q1, Q3]	14.0 [5.00, 21.0]	14.0 [7.75, 21.0]	14.0 [7.00, 21.0]
Missing	46 (1.6%)	60 (2.0%)	40 (1.3%)
**Birth year**			
2010-2013	1,057 (37.0%)	1,190 (39.8%)	1,233 (40.1%)
2014-2017	990 (34.7%)	1,089 (36.4%)	1,221 (39.8%)
2018-2020	806 (28.3%)	709 (23.7%)	617 (20.1%)

© 2026 Aveline A et al. *JAMA Network Open*

3c. Ozone Dominant Air Quality Index

	Low (N=2,823)	Medium (N=2,988)	High (N=3,101)
**Gestational age at birth (weeks)**			
22-23	194 (6.9%)	238 (8.0%)	181 (5.8%)
24-26	1,350 (47.8%)	1,370 (45.9%)	1,427 (46.0%)
27-28	1,279 (45.3%)	1,380 (46.2%)	1,493 (48.1%)
**Sex**			
Male	1,486 (52.6%)	1,606 (53.7%)	1,667 (53.8%)
Female	1,337 (47.4%)	1,382 (46.3%)	1,434 (46.2%)
**Birthweight**			
Median [Q1, Q3]	900 [730, 1,090]	890 [710, 1,090]	900 [730, 1,090]
**Small for gestational age**			
Not SGA	2,562 (90.8%)	2,724 (91.2%)	2,809 (90.6%)
SGA	261 (9.2%)	264 (8.8%)	292 (9.4%)
**Snap II Score**			
Median [Q1, Q3]	14.0 [7.00, 21.0]	14.0 [7.00, 21.0]	14.0 [7.00, 21.0]
Missing	41 (1.5%)	62 (2.1%)	43 (1.4%)
**Birth year**			
2010-2013	1,081 (38.3%)	1,110 (37.1%)	1,289 (41.6%)
2014-2017	942 (33.4%)	1,187 (39.7%)	1,171 (37.8%)
2018-2020	800 (28.3%)	691 (23.1%)	641 (20.7%)

© 2026 Aveline A et al. *JAMA Network Open*

3d. Temperature Index

	Low (N=2,898)	Medium (N=2,918)	High (N=3,096)
**Gestational age at birth (weeks)**			
22-23	168 (5.8%)	225 (7.7%)	220 (7.1%)
24-26	1,363 (47.0%)	1,319 (45.2%)	1,465 (47.3%)
27-28	1,367 (47.2%)	1,374 (47.1%)	1,411 (45.6%)
**Sex**			
Male	1,507 (52.0%)	1,552 (53.2%)	1,700 (54.9%)
Female	1,391 (48.0%)	1,366 (46.8%)	1,396 (45.1%)
**Birthweight**			
Median [Q1, Q3]	900 [730, 1,090]	890 [710, 1,090]	893 [719, 1,080]
**Small for gestational age**			
Not SGA	2,620 (90.4%)	2,648 (90.7%)	2,827 (91.3%)
SGA	278 (9.6%)	270 (9.3%)	269 (8.7%)
**Snap II Score**			
Median [Q1, Q3]	14.0 [5.00, 21.0]	14.0 [7.00, 21.0]	14.0 [7.00, 21.0]
Missing	39 (1.3%)	52 (1.8%)	55 (1.8%)
**Birth year**			
2010-2013	779 (26.9%)	1,136 (38.9%)	1,565 (50.5%)
2014-2017	1,383 (47.7%)	1,011 (34.6%)	906 (29.3%)
2018-2020	736 (25.4%)	771 (26.4%)	625 (20.2%)

© 2026 Aveline A et al. *JAMA Network Open*

eTable 4: Results from the sensitivity analysis using multiple imputation

Outcome = Survival without major morbidity

Index	Tertiles Contrasted	Risk difference (%)	Adjusted Odds Ratio1 (95% CI)	p-value for adjusted analysis
**Greenness**	Highest-Lowest	1.5	1.14 (1.00-1.31)	0.06
	Moderate-Lowest	1.5	1.14 (1.02-1.29)	0.02
	Highest-Moderate	0.0	1.00 (0.88-1.14)	1.00
**$PM_{2.5}$ dominant index (PM$_{2.5}$, NO$_2$, Smoke)**	Highest-Lowest	-0.2	0.98 (0.85-1.13)	0.94
	Moderate-Lowest	0.2	1.02 (0.89-1.18)	0.91
	Highest-Moderate	-0.5	0.96 (0.84-1.08)	0.68
**Ozone dominant index**	Highest-Lowest	-2.9	0.77 (0.67-0.88)	0.04
	Moderate-Lowest	-1.6	0.87 (0.76-1.00)	0.04
	Highest-Moderate	-1.3	0.88 (0.77-1.01)	0.08
**Temperature**	Highest-Lowest	1.8	1.24 (1.10-1.40)	0.0001
	Moderate-Lowest	2.3	1.19 (1.06-1.35)	0.002
	Highest-Moderate	0.4	1.04 (0.92-1.17)	0.73

© 2026 Aveline A et al. *JAMA Network Open*

eFigure 1: Directed acyclic graph summarizing the causal relationships associated with the exposure and outcome

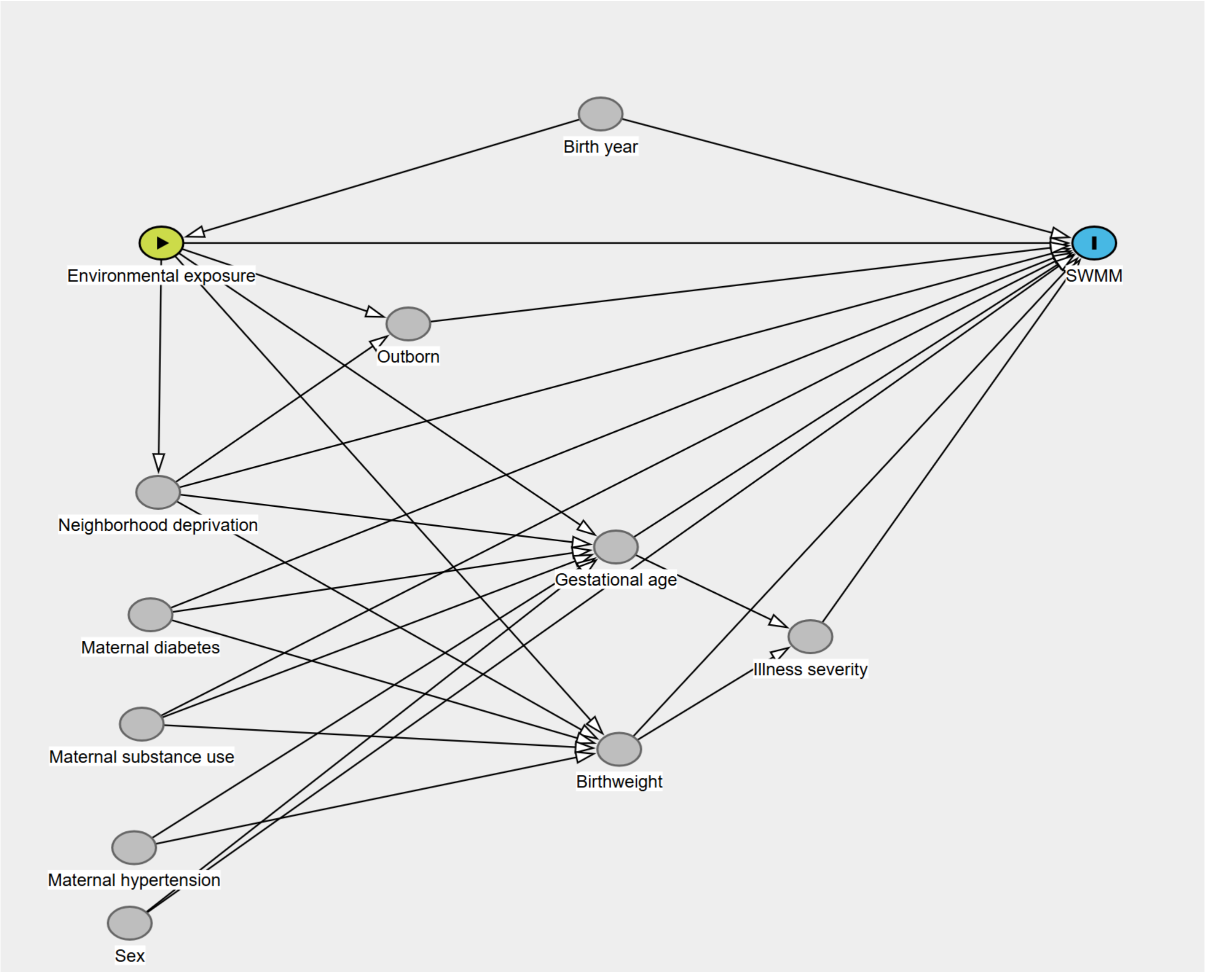

© 2026 Aveline A et al. *JAMA Network Open*

eFigure 2: Rates of survival without major morbidity stratified by tertiles of the exposure indices

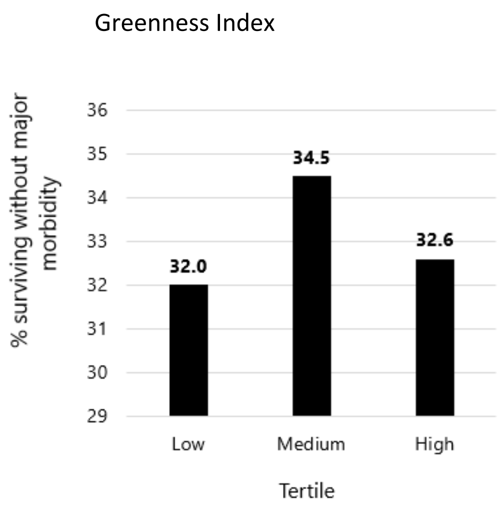

Lower values on the greenness index represent in-utero exposure to a less green environment.

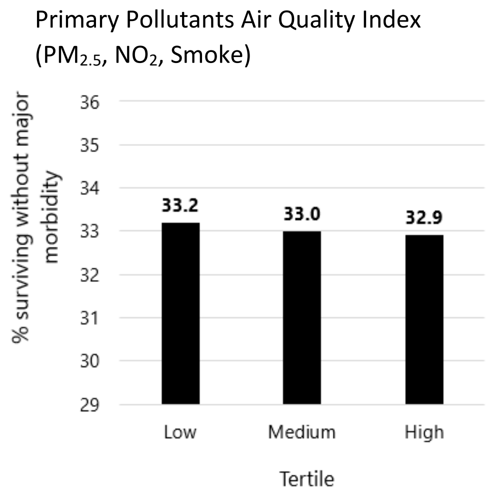

Lower values on the primary pollutants index are associated with low levels of $PM_{2.5}$ and NO_2 and high levels of smoke

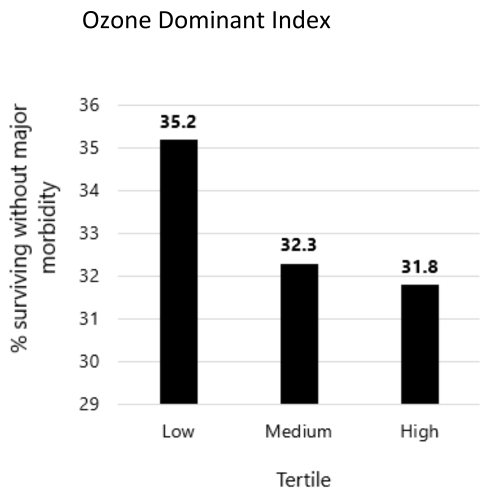

Lower values on the air pollution index are predominantly associated with lower levels of ozone.

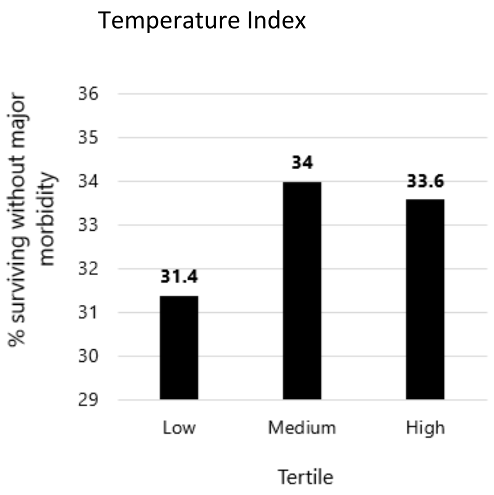

Lower values on the temperature index are associated with: low maximum monthly temperatures, low minimum monthly temperatures or more days with temperatures below -15°

[1] Adjusted for gestational age at birth, inborn/outborn status, birthweight, birth epoch, maternal diabetes, maternal antenatal drug use, maternal antenatal hypertension, infant small for gestational age, sex, SNAP-II and neighborhood risk indices in tertiles.

© 2026 Aveline A et al. *JAMA Network Open*
