# Supplementary material for: Antepartum Exposure to Greenness, Air Pollution, and Temperature and Outcomes of Preterm Infants
Source: JAMA Netw Open. 2026 Feb 26;9(2):e260102. doi: 10.1001/jamanetworkopen.2026.0102 (PMC12947023; doi:10.1001/jamanetworkopen.2026.0102)
Supplement: Supplement 2. — Nonauthor Collaborators [file jamanetwopen-e260102-s002.pdf]

*Indicates required information. Only first name, last name, and suffix will appear in PubMed.

*Group Name(s): CNN Investigators							
***First Name and Middle Initial(s)**	***Last Name**	***Suffix (eg, Jr, III)**	Academic Degrees	Institution	Location (city, state/province, country)	Role or Contribution, eg, chair, principal investigator	Group (if more than 1 Group listed in the byline) and/or Subgroup (eg, Steering Committee)
Thevanisha	Pillay		MD, MSc	Victoria General Hospital, Victoria, British Columbia	Canada	Site investigator	Canadian Neonatal Network
Jonathan	Wong		MD	British Columbia Women's Hospital, Vancouver, British Columbia	Canada	Site investigator	Canadian Neonatal Network
Miroslav	Stavel		MD	Royal Columbian Hospital, New Westminster, British Columbia	Canada	Site investigator	Canadian Neonatal Network
Rebecca	Sherlock		MD	Surrey Memorial Hospital, Surrey, British Columbia	Canada	Site investigator	Canadian Neonatal Network
Ayman	Abou Mehrem		MD	Foothills Medical Centre, Calgary, Alberta	Canada	Site investigator	Canadian Neonatal Network
Jennifer	Toye		MD	Royal Alexandra Hospital and University of Alberta Hospital, Edmonton, Alberta	Canada	Site investigator	Canadian Neonatal Network
Joseph	Ting		MD	Royal Alexandra Hospital and University of Alberta Hospital, Edmonton, Alberta	Canada	Site investigator	Canadian Neonatal Network
Carlos	Fajardo		MD	Alberta Children's Hospital, Calgary, Alberta	Canada	Site investigator	Canadian Neonatal Network
Andrei	Harabor		MD	Regina General Hospital, Regina, Saskatchewan	Canada	Site investigator	Canadian Neonatal Network
Lannae	Strueby		MD	Jim Pattison Children's Hospital, Saskatoon, Saskatchewan	Canada	Site investigator	Canadian Neonatal Network
Mary	Seshia		MD	Winnipeg Health Sciences Centre, Winnipeg, Manitoba	Canada	Site investigator	Canadian Neonatal Network
Deepak	Louis		MBChB	Winnipeg Health Sciences Centre, Winnipeg, Manitoba	Canada	Site investigator	Canadian Neonatal Network

*Indicates required information. Only first name, last name, and suffix will appear in PubMed.

***First Name and Middle Initial(s)**	***Last Name**	***Suffix (eg, Jr, III)**	Academic Degrees	Institution	Location (city, state/province, country)	Role or Contribution, eg, chair, principal investigator	Group (if more than 1 Group listed in the byline) and/or Subgroup (eg, Steering Committee)
Chelsea	Ruth		MD	St. Boniface General Hospital, Winnipeg, Manitoba	Canada	Site investigator	Canadian Neonatal Network
Ann	Yi		MD	St. Boniface General Hospital, Winnipeg, Manitoba	Canada	Site investigator	Canadian Neonatal Network
Amit	Mukerji		MD	Hamilton Health Sciences Centre, Hamilton, Ontario	Canada	Site investigator	Canadian Neonatal Network
Kevin	Coughlin		MD	London Health Sciences Centre, London, Ontario	Canada	Site investigator	Canadian Neonatal Network
Sajit	Augustine		MD, MSc	Windsor Regional Hospital, Windsor, Ontario	Canada	Site investigator	Canadian Neonatal Network
Kyong-Soon	Lee		MD	Hospital for Sick Children, Toronto, Ontario	Canada	Site investigator	Canadian Neonatal Network
Eugene	Ng		MD, MSc	Sunnybrook Health Sciences Centre, Toronto, Ontario	Canada	Site investigator	Canadian Neonatal Network
Brigitte	Lemyre		MD	The Ottawa Hospital, Ottawa, Ontario and Children’s Hospital of Eastern Ontario, Ottawa, Ontario	Canada	Site investigator	Canadian Neonatal Network
Faiza	Khurshid		MD	Kingston General Hospital, Kingston, Ontario	Canada	Site investigator	Canadian Neonatal Network
Victoria	Bizgu		MD	Jewish General Hospital, Montréal, Québec	Canada	Site investigator	Canadian Neonatal Network
Keith	Barrington		MD	Hôpital Sainte-Justine, Montréal, Québec	Canada	Site investigator	Canadian Neonatal Network
Anie	Lapointe		MBChB	Hôpital Sainte-Justine, Montréal, Québec	Canada	Site investigator	Canadian Neonatal Network
Guillaume	Ethier		MD	Hôpital Sainte-Justine, Montréal, Québec	Canada	Site investigator	Canadian Neonatal Network
Christine	Drolet		MD	Centre Hospitalier Universitaire de Québec, Sainte Foy, Québec	Canada	Site investigator	Canadian Neonatal Network

*Indicates required information. Only first name, last name, and suffix will appear in PubMed.

*First Name and Middle Initial(s)	*Last Name	*Suffix (eg, Jr, III)	Academic Degrees	Institution	Location (city, state/province, country)	Role or Contribution, eg, chair, principal investigator	Group (if more than 1 Group listed in the byline) and/or Subgroup (eg, Steering Committee)
Marco	Zeid		NNP	Montreal Children's Hospital at McGill University Health Centre, Montréal, Québec	Canada	Site investigator	Canadian Neonatal Network
Marie	St-Hilaire		MD	Hôpital Maisonneuve-Rosemont, Montréal, Québec	Canada	Site investigator	Canadian Neonatal Network
Valerie	Bertelle		MD	Centre Hospitalier Universitaire de Sherbrooke, Sherbrooke, Québec	Canada	Site investigator	Canadian Neonatal Network
Edith	Masse		MD	Centre Hospitalier Universitaire de Sherbrooke, Sherbrooke, Québec	Canada	Site investigator	Canadian Neonatal Network
Paloma	Costa		MD	Moncton Hospital, Moncton, New Brunswick	Canada	Site investigator	Canadian Neonatal Network
Hala	Makary		MD	Dr. Everett Chalmers Hospital, Fredericton, New Brunswick	Canada	Site investigator	Canadian Neonatal Network
Ahmad	Aziz		MD	Dr. Everett Chalmers Hospital, Fredericton, New Brunswick	Canada	Site investigator	Canadian Neonatal Network
Gabriela	de Carvalho Nunes		MD	Saint John Regional Hospital, Saint John, New Brunswick	Canada	Site investigator	Canadian Neonatal Network
Wissam	Alburaki		MD	Saint John Regional Hospital, Saint John, New Brunswick	Canada	Site investigator	Canadian Neonatal Network
Jo-Anna	Hudson		MD	Janeway Children's Health and Rehabilitation Centre, St. John's, Newfoundland	Canada	Site investigator	Canadian Neonatal Network
Jehier	Afifi		MD	IWK Health Centre, Halifax, Nova Scotia	Canada	Site investigator	Canadian Neonatal Network
Andrzej	Kajetanowicz		MB BCh, MSc	Cape Breton Regional Hospital, Sydney, Nova Scotia	Canada	Site investigator	Canadian Neonatal Network
Bruno	Piedboeuf		MD	Centre Hospitalier Universitaire de Québec, Sainte Foy, Québec	Canada	Site investigator	Canadian Neonatal Network

Supplemental Online Content: Nonauthor Collaborators

*Indicates required information. Only first name, last name, and suffix will appear in PubMed.

***First Name and Middle Initial(s)**	***Last Name**	***Suffix (eg, Jr, III)**	Academic Degrees	Institution	Location (city, state/province, country)	Role or Contribution, eg, chair, principal investigator	Group (if more than 1 Group listed in the byline) and/or Subgroup (eg, Steering Committee)
**Group name: Canadian Urban Environmental Health Research Consortium**							
