# Supplementary material for: Antepartum Exposure to Greenness, Air Pollution, and Temperature and Outcomes of Preterm Infants
Source: JAMA Netw Open. 2026 Feb 26;9(2):e260102. doi: 10.1001/jamanetworkopen.2026.0102 (PMC12947023; doi:10.1001/jamanetworkopen.2026.0102)
Supplement: Supplement 3. — Data Sharing Statement [file jamanetwopen-e260102-s003.pdf]

Data Sharing Statement

Aveline. Antepartum Exposure to Greenness, Air Pollution, and Temperature and Outcomes of Preterm Infants. *JAMA Netw Open*. Published February 26, 2026.
doi:10.1001/jamanetworkopen.2026.0102

Data
Data available: No

Additional Information
Explanation for why data not available: We do not have appropriate permission from individual renters or patients to allow for data sharing.
